# Electroretinogram responses in myopia: a review

**DOI:** 10.1007/s10633-021-09857-5

**Published:** 2021-11-17

**Authors:** Satish Kumar Gupta, Ranjay Chakraborty, Pavan Kumar Verkicharla

**Affiliations:** 1 Myopia Research Lab, Prof. Brien Holden Eye Research Centre, Brien Holden Institute of Optometry and Vision Sciences, Kallam Anji Reddy Campus, L V Prasad Eye Institute, Hyderabad, India; 2 Caring Futures Institute, College of Nursing and Health Sciences, Optometry and Vision Science, Flinders University, Adelaide, South Australia Australia

**Keywords:** Axial length, Electroretinogram (ERG), Myopia, Neurons, Neurophysiology, Retina

## Abstract

The stretching of a myopic eye is associated with several structural and functional changes in the retina and posterior segment of the eye. Recent research highlights the role of retinal signaling in ocular growth. Evidence from studies conducted on animal models and humans suggests that visual mechanisms regulating refractive development are primarily localized at the retina and that the visual signals from the retinal periphery are also critical for visually guided eye growth. Therefore, it is important to study the structural and functional changes in the retina in relation to refractive errors. This review will specifically focus on electroretinogram (ERG) changes in myopia and their implications in understanding the nature of retinal functioning in myopic eyes. Based on the available literature, we will discuss the fundamentals of retinal neurophysiology in the regulation of vision-dependent ocular growth, findings from various studies that investigated global and localized retinal functions in myopia using various types of ERGs.

## Introduction

The prevalence of myopia is on the rise worldwide since a few decades [1]. Various meta-analysis studies have predicted that approximately 5 billion of the global population may develop myopia by the year 2050, with around 1 billion of them having high myopia [2, 3]. The increasing prevalence of myopia and its associated sight-threatening risks [3–9] make myopia a major public health concern [10–12] and demands investigation into the fundamentals of eye growth regulation. The ocular stretching in myopia is associated with several structural and functional changes in posterior segment of the eye [13, 14]. Recent research highlights the role of retinal signaling in ocular growth, and various studies have investigated the electrophysiological responses in different types of refractive errors. This review is aimed to provide a summary of research work on electroretinogram (ERG) responses in myopia and their implications in understanding the nature of retinal functioning in myopic eyes.

## Retinal development, photo-transduction, and regulation of vision-dependent ocular growth

The development of neural retina usually begins on day 26 of gestation [15], where the inner neural ectoderm divides into 3–4 layers of cells [16]. By week 12, the retinal layers start to form, with the inner neuroblastic layer giving rise to the ganglion, amacrine, and Muller cells creating the inner retina [17]. Similarly, the outer neuroblastic layer gives rise to photoreceptors (rods and cones), bipolar and horizontal cells forming the outer retina [17]. By the end of week 14, the ganglion cells migrate away from the fovea toward retinal periphery and cone photoreceptors migrate toward the fovea [16, 18].

Retina being the only photo-sensitive neural layer in the eye [19], incorporates about 55 types of structurally and functionally specific neurons [20, 21] including photoreceptors, bipolar cells, ganglion cells, horizontal cells, and amacrine cells [22, 23]. The distinct arrangement of these neurons from outer to inner retina forms a complex circuit to capture the photons of light from an object [24–26]. These photons are converted into electrical/neuronal signals by the photoreceptors with the help of visual pigments present in them by a process called “photo-transduction” [26, 27]. The retinal photoreceptors, through synapses with retinal bipolar cells, transmit signals to the retinal ganglion cells. Upon activation, the axons of retinal ganglion cells carry neuronal signals to the brain via optic nerve for visual perception [28]. Evidence from animal studies suggests that both inner and outer retina may influence the detection of optical defocus and signaling for the corresponding development of ocular growth [19, 29–34].

Several animal species including chicks [35–44], squid [45], tree shrews [33, 46], monkeys [47–55], marmosets [56], guinea pigs [57], kittens [58–60], mice [61–63], and also humans [64–68] are capable of identifying the sign and magnitude of retinal image defocus and make compensatory alterations in ocular growth [69–74]. Evidence from the experiments conducted on animal models indicates that the absence of input from the accommodative system (cycloplegia, ciliary nerve section, or damage to the Edinger–Westphal nucleus) [31, 39] or higher visual center (optic nerve section) [75] does not influence the ocular response to imposed form-deprivation [38, 75], or optical defocus [31, 39, 42, 75, 76], suggesting that the visual mechanisms regulating the refractive development are primarily localized at the retina.

Given that the fovea provides the best visual acuity (largely attributed to cone signaling) [77, 78], it was traditionally assumed that cone pathways may have a greater influence on visual signaling for refractive development [70, 79]. However, as the foveal area corresponds to only a small part of the visual field, it is reasonable to assume that the peripheral retinal areas might also be important in driving refractive status. There is growing evidence involving animal models indicating the presence of ocular growth pathways mediated by signals from the peripheral retina. The normal response to a) form-deprivation in monkeys treated with laser ablation at the cone-rich fovea [52], b) similar myopic responses in monkeys with form-deprivation [48, 53] and hyperopic defocus [51, 54] imposed on the rod-dominated peripheral regions or the entire visual field, and c) recent work on Gnat1^*−/−*^ mice with rod dysfunction [63] indicate that the peripheral rod pathways may be equally critical for visually guided eye growth. Blocking the functions of photoreceptors [19, 80], ON and OFF pathways [60, 81–83] by pharmacological means (neurotoxins) [30–32, 34] or genetic means (such as in mouse models) [84–88] is known to affect both normal refractive development and response to form-deprivation myopia (FDM) showing the importance of various retinal neurons, neuronal pathways, and neurotransmitters in the refractive development of eye [89, 90]. Overall, these studies support the hypothesis that refractive development occurs through a cascade of local and regionally selective mechanisms in the retina [55, 70, 73, 74, 79].

## Electroretinogram (ERG)

The retinal function can be assessed by electrophysiological tests that study the electrical properties of the biological cells and tissues, driven by the flow of ions (ion current) [26, 91–93]. Of various electrophysiological tests, the electroretinogram (ERG) with the standard protocol by the International Society for Clinical Electrophysiology of Vision (ISCEV) is widely used to determine the global and localized retinal responses [94–97]. When a bright flash of light illuminates the retina, changes in membrane potentials across the neuronal and non-neuronal retinal cells simultaneously and instantaneously with a high temporal resolution (milliseconds) [94–97] give rise to an extracellular current, which forms the basis of ERG [98–100]. Hence, the ERG test provides a unique opportunity to investigate changes in retinal electrical activity to several inherited and acquired retinal diseases [94–97] and several disorders or ocular conditions including refractive errors [101]. The most commonly used ERG techniques are full-field flash ERG (ffERG), multifocal ERG (mfERG), and pattern ERG (PERG).

## Full-field flash electroretinogram (ffERG) and its responses in myopia

**Fig. 1:**
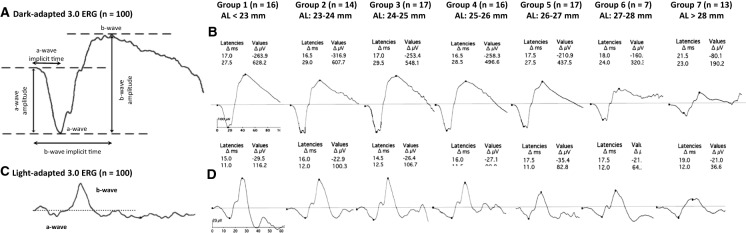
Normal waveforms and parameters of **A** dark‐adapted 3.0 ERG and **C** light‐adapted 3.0 ERG of full‐field electroretinogram (ffERG). Average ffERG responses from **B** dark‐adapted 3.0 ERG and **D** light‐adapted 3.0 ERG from 100 eyes with axial length (AL) ranging from 21.79 to 30.55 mm and spherical equivalent refractive error (SER) ranging from + 0.50 to − 18.00 D. All participants were divided into seven different groups based on their mean AL: Group 1 (22.40 mm), Group 2 (23.10 mm), Group 3 (24.26 mm), Group 4 (25.51 mm), Group 5 (26.34 mm), Group 6 (27.5 mm), and Group 7 (29.55 mm). The two values listed for each group under latencies and values, respectively, indicate the change (Δ) in latencies (Δ ms) and amplitudes (Δ µV) of a ffERG a-wave (negative values) and b-wave (positive values). Adapted with permission from Sachidanandam et al. (2017) [109]

A flash ERG measures the average response of retinal cells from a relatively broad retinal region to a full-field luminance stimulation [94]. By varying the background illumination, the light- or dark-adapted state of the eye, and the intensity of stimulus flash, one can elicit and isolate responses from different retinal cells. A standard ERG waveform is usually biphasic, with an initial cornea-negative response (a-wave), followed by a cornea-positive response (b-wave), and an additional slower positive wave or c-wave (Fig. 1a: scotopic and Fig. 1c: photopic) [97, 102]. In general, electrical activity in the photoreceptors, ON-bipolar cells, and retinal pigment epithelium initiate the a-wave [103], b-wave [104], and c-wave [102], respectively. The oscillatory potentials (OPs) that indicate the activity of amacrine cells in inner retina are represented by a small high-frequency wavelet component on the ascending limb of b-waves [94].

The results from several studies that investigated ffERG responses in myopia are given in Table 1. The overall incidence of abnormal electrophysiological findings in myopes younger than 18 years of age was reported to be 29%, with a higher proportion of ERG abnormalities reported in higher ametropias (spherical equivalent refractive error, SER worse than  ± 6.00 D; 52%) compared to individuals with emmetropia (SER:  − 0.75 D to + 1.50 D; 26%) or low ametropia (SER lower than ± 6.00 D) [101].

**Table 1:** Summary of studies on full-field flash ERG (ffERG) and myopia

References	Participants	Outcomes
Blach et al. [119]	25 emmetropes and 30 high myopes with degenerative fundus changes	Increased a-wave and reduced b-wave amplitude with increased degree of myopia
Malik et al.[112]	43 myopes with degenerative fundus changes and 37 myopes with normal fundus	Reduced a- and b-wave amplitudes as the degree of myopia increased. Decreased ffERG responses in the eyes with degenerative fundus changes, irrespective of the degree of myopia
Perlman et al. [107]	31 high hypermetropes (> + 5.00 D), 7 high myopes (< -6.00 D), and 7 unilateral or bilateral aphakics	Reduced scotopic a-and b-wave and photopic b-wave amplitudes in high myopes
Ishikawa et al. [120]	66 high myopes and 76 emmetropes	Reduced a-and b-wave amplitudes in tigroid fundus. Reduced a- and b-wave, and OPs' amplitudes and increased implicit time in posterior staphyloma involving the macula
Westall et al. [108]	33 high myopes (− 6.00 to − 14.50 D), 8 mild myopes (− 3.00 to − 5.00 D), and 19 small SER (+ 0.75 to − 2.75 D)	Reduced rod-cone a-and b-wave, cone b-wave, and OPs' amplitudes in high myopes, which was proportional to increased AL
Yoshii et al. [113]	14 emmetropes (− 0.50 to − 3.50 D) and 16 high myopes (− 7.00 to − 11.50 D)	Reduced nonlinear component of the ERG amplitudes from the posterior pole of the fundus in high myopes
Flitcroft et al. [101]	15 high myopes (≤ − 6.00 D), 19 low myopes (− 0.75 to − 6.00 D), 35 emmetropes (− 0.75 to + 1.50 D), 44 low hyperopes (+ 1.50 to + 6.00 D), and 10 high hyperopes (≥ + 6.00 D)	Abnormal ffERG responses in high ammetropia
Shamshinova et al. [111]	46 myopes with moderate-to-high congenital myopia	Reduced b-wave amplitude with increased degree of myopia and AL
Kader et al. [106]	40 emmetropes (± 0.25 D), 20 mild myopes (− 0.50 to − 3.00 D), 28 moderate myopes (− 3.25 to − 6.00 D), 40 high myopes (− 6.25 to − 15.00 D), and 40 pathological myopes (− 7.00 to − 22.00 D with 7 posterior staphyloma)	Reduced scotopic, photopic, and combined b-wave, OPs', and 30 Hz flicker amplitudes as well as delayed latencies in high myopes, which was proportional to increased AL
Wang et al. [117]	64 early-onset high myopes and 20 late-onset high myopes	Reduced scotopic b-wave, photopic a- and b-wave, and combined a- and b-wave amplitudes in early-onset high myopes
Koh et al. [110]	32 myopes (≤ − 6.00 D)	Reduced scotopic b-wave, photopic a- and b-wave, and 30 Hz flicker b-wave amplitudes with increased degree of myopia and AL
Sachidanandam et al. [109]	100 eyes with axial length ranging from 21.79 to 30.55 mm and SER ranging from + 0.50 to − 18.00 D	Reduced both scotopic and photopic a- and b-wave amplitudes and minimal delayed corresponding IT with increased AL
Wan et al. [122]	19 emmetropes (± 0.25 D), 18 low myopes (− 0.50 to − 3.00 D), 23 moderate myopes (− 3.25 to − 6.00 D), and 16 high myopes (≤ − 6.25 D)	Increased scotopic a- and b-wave amplitudes as well as rod-driven OPs' peak frequency with increased degree of myopia

*AL* Axial length, *ERG* Electroretinogram, *ffERG* full-field flash electroretinogram, *IT* implicit time, *OPs* oscillatory potentials, *SER* spherical equivalent refractive error

**Fig. 2:**
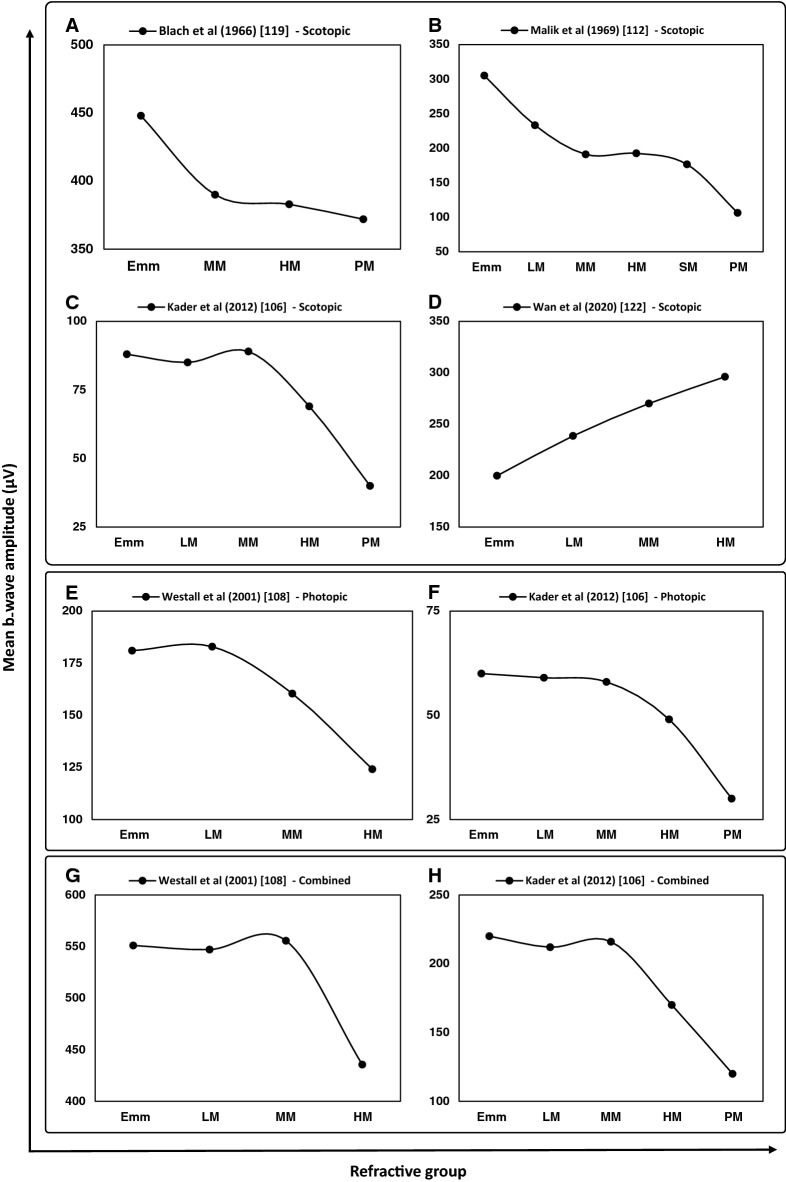
Mean b-wave amplitudes for scotopic (**A–D**), photopic (**E**, **F**), and combined response (**G**, **H**) of full-field flash ERG (ffERG) reported by each study in emmetropia (Emm) and various grades of myopia (*LM*: low myopia, *MM*: moderate myopia, *HM*: high myopia, *SM*: severe myopia, and *PM*: pathological myopia)

Since the first report of conventional ERG in myopes by Karpe in 1945 [105], various studies have reported impairment of retinal function in myopia. Several studies reported a significant reduction of b-wave amplitude in myopia that closely correlated with the degree of myopia and the axial length of eye [106–115]. For every 1-mm increase in axial length, the dark-adapted 3.0 ERG showed a reduction of 15.7 μV and 23.4 μV in a-wave and b-wave amplitude, respectively, in an absence of a myopic retinal degeneration [109]. Significant differences in both a- and b-wave amplitudes of ffERG have been reported in high myopia (SER:  − 6.00 D to − 14.50 D), moderate myopia ( − 3.00 D to − 5.00), and low refractive error (+ 0.75 D to − 2.75 D) [108, 110] with a significant reduction in the b-wave amplitude under both scotopic (Fig. 1b) and photopic (Fig. 1d) conditions for individuals with high myopia (Fig. 2) [106, 107, 110, 111]. Scotopic responses (dark-adapted 3.0 ERG) were, however, reported to be more significantly affected than the photopic responses (light-adapted 3.0 and 30 Hz flicker ERG) [109, 110].

Studies have shown significantly lower short (S), long (L) and middle (M) wavelength-sensitive cone ERG b-wave amplitudes in high myopic eyes than the non-myopic eyes [116]. Significant reduction of cone and rod responses (mostly cones) in individuals with early-onset of high myopia (onset age, ≤ 5 years) than those with late-onset of high myopia (onset age, 12.4 ± 2.5 years) suggests that cone-rod dysfunction may be a sign for early onset of high myopia [117, 118]. The ffERG [119] and focal macular ERG [120] findings in pathologic myopes (presence of myopic retinal degeneration caused by progressive stretching and thinning of posterior segment of eye due to excessive axial length elongation which can result in reduced best-corrected visual acuity [12, 121]) showed a significant reduction in the amplitude of a- and b-wave in high myopic eyes with tigroid fundus appearance when compared to emmetropic eyes, whereas the implicit time was within the normal range. Similarly, there was a significant reduction in the amplitude of a-wave, b-wave, and OPs and delay in implicit time in high myopia with posterior staphyloma involving the macula compared to early myopia with tigroid fundus [120]. The reduced amplitude with normal implicit time in high myopia with tigroid fundus was related to a significant reduction in the macular cone density (focal macular ERG), which is considered to be an early macular change in high myopia [119, 120]. Furthermore, it was suggested that the reduced amplitude with delayed latency in high myopia associated with macular pathologies such as posterior staphyloma involving the macula could further reduce macular cone photoreceptors [120]. Likewise, chorioretinal vascular changes, retinal pigment epithelium degeneration, and receptor changes found in degenerative myopia may also play a major role in altering the ERG responses [119].

In contrast, Wan et al. (2020) recently reported an increase in the amplitude of a- and b-wave of the scotopic/dark-adapted 3.0 ERG (combined responses arising from the photoreceptors and ON-bipolar cells of both the rod and cone systems; rod-dominated) with the degree of myopia [122]. In addition, the average peak frequency of the rod-driven dark-adapted OPs arising from amacrine cells and the inner plexiform layer also showed a significant positive correlation with the magnitude of myopia [122]. The authors argued that these inconsistencies in comparison with other studies reflected the composition of the participants in their study, being young adults without any sign of pathological myopia (i.e., myopic retinal degenerations). In addition, the responses obtained from previous studies reflected combined contributions of the rod- and cone-driven OPs, interfering with each other [123–126], while Wan et al. isolated the rod-driven OPs by subtracting the light-adapted ERG from the dark-adapted ERG [127, 128]. Their findings indicate an alteration in the rod and ON-bipolar cell function in myopia, with minimal effect on the cone system. It is hypothesized that changes in the rod-mediated retinal function may be related to the changes in the retinal dopaminergic pathways (dopamine D2 receptors) [122, 129]. The variation in the dark-adapted 3.0 ERG OP amplitudes in myopes indicates an imbalance of the 'ON' and 'OFF' retinal activity, which may be associated with the development of myopia and its progression [130].

## Multifocal electroretinogram (mfERG) and its responses in myopia

A conventional ffERG measures the global electrical response of the entire retina, but it does not provide a localized response [131]. A mfERG is applicable for objectively studying local retinal health as well as characterizing and monitoring focal retinal lesions in various pathological conditions [132–134]. The mfERG uses a specific hexagonal stimulus pattern to obtain a topographic map of retinal electrophysiological activity over a restricted retinal region (~ 40–50°), unlike ffERG, that reflects light-induced electrical activity from almost the entire retina [95]. This specific hexagonal pattern stimulus illuminates the retina using a pseudo-random binary m-sequence algorithm and gives rise to a continuously recorded signal from individual retinal locations [95]. All the localized responses can be averaged to compare quadrants, hemi-retinal areas, normal and abnormal regions of the two eyes, or successive rings from center to periphery [95]. Routinely, the stimulus pattern (array) with 61 or 103 hexagons is used within a field diameter of 40–50° (20–25° radius from the point of fixation to the edge of display) [95]. In the case of 61 hexagons, they are grouped from center to periphery into five rings (R1–R5), where R1 is the central ring and R5 is the peripheral ring. The approximate eccentricity from R1 to R5 is < 2°, 2–5°, 5–10°, 10–15°, and > 15° (~ 23°), respectively [135]. A similar grouping for 103 hexagons display would have a total of six rings within the same field diameter.

**Fig. 3:**
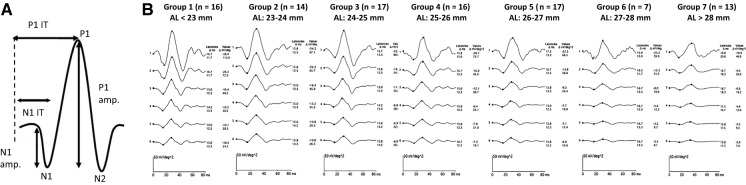
**A** Normal multifocal electroretinogram (mfERG) waveform and parameters. **B** Average six mfERG ring responses from 100 eyes with axial length (AL) ranging from 21.79 to 30.55 mm and spherical equivalent refractive error (SER) ranging from + 0.50 to − 18.00 D. All participants were divided into seven different groups based on their mean AL: Group 1 (22.40 mm), Group 2 (23.10 mm), Group 3 (24.26 mm), Group 4 (25.51 mm), Group 5 (26.34 mm), Group 6 (27.5 mm), and Group 7 (29.55 mm). The two values listed for each group under latencies and under values, respectively, indicate the change (Δ) in latencies (Δ ms) and amplitude density (Δ nV/deg^2^) of a mfERG N1 (negative values) and P1 (positive values) wave. Adapted with permission from Sachidanandam et al. (2017) [109]

A typical mfERG waveform (also called the first-order response, or first-order kernel) is analogous to the conventional ffERG response as it is biphasic, with an initial negative component (N1), followed by a positive peak (P1) (Fig. 3a) [95]. There is another second negative deflection (N2) after the positive peak (P1). In humans, the N1 component primarily originates from the cone photoreceptors with minimal contribution from ON- and OFF-cone bipolar cells, the P1 component arises from the activity of ON- and OFF-cone bipolar cells, and the N2 component is derived from inner retinal cells (amacrine and ganglion cells) [94, 95, 136, 137].

**Fig. 4:**
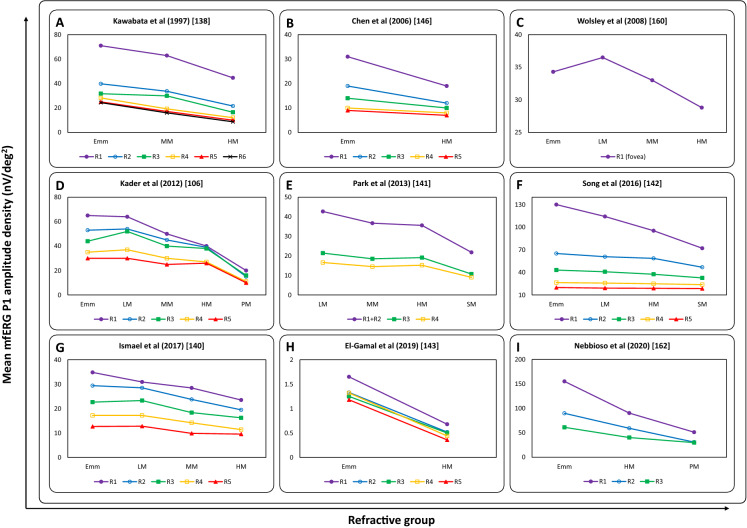
Mean mfERG P1 amplitude density (nV/deg^2^) at each ring (R1: filled purple circles, R2: unfilled blue circles, R3: filled green squares, R4: unfilled orange squares, R5: filled red triangles, and R6: black cross) reported by each study in emmetropia (Emm) and various grades of myopia (LM: low myopia, MM: moderate myopia, HM: high myopia, SM: severe myopia, and PM: pathological myopia)

The results from several studies that investigated mfERG responses in myopia are given in Table 2. Previous investigations on early changes in retinal function using the first-order kernel responses in low, medium, and high myopes showed a significant reduction of N1, P1 [106, 109–111, 138–143], and N2 amplitude density [139] with a greater effect on P1 amplitudes than N1 amplitudes (Fig. 3b and Fig. 4) [109, 143, 144]. The reduction in N1 and P1 amplitude density and significant delay in corresponding latencies [145, 146] in all rings, as well as four retinal quadrants, was significantly correlated with axial length and the degree of myopic refractive error [106, 109–111, 138–144]. The delayed implicit time was attributed to the possible altered synaptic transmission between the ON- and OFF-bipolar cells or structural changes in the inner plexiform layer of retina [147].

**Table 2:** Summary of studies on multifocal ERG (mfERG) and myopia

References	Participants	Outcomes
Kawabata et al. [138]	10 emmetropes/low myopes (+ 1.00 to − 3.00 D), 10 moderate myopes (− 3.25 to − 6.00 D), 10 high myopes (≤ − 6.25 D)	Reduced N1, P1 amplitudes and delayed corresponding latencies with increased degree of myopia and retinal eccentricity
Sun et al. [148]	20 emmetropes, 20 mild myopes, 20 moderate myopes, and 20 high myopes	Reduced N1, P1, N2 amplitudes and corresponding response density with increased degree of myopia and retinal eccentricity
Chan et al. [158]	30 subjects with axial length ranging from 23.72 to 28.13 mm and SER ranging from 0.00 to − 10.50 D	Reduced P1 amplitude in the central (R1) and reduced N1, P1 amplitudes in the paracentral region (R3) with increased degree of myopia and AL. The mfERG amplitude reduced by about 6–10% per 1-mm increase in AL
Luu et al. [139]	104 children and 31 adults with SER ranging from 0.00 to − 10.00 D	Reduced N1, P1, N2 amplitudes and delayed corresponding IT with increased degree of myopia in adults
Luu et al. [149]	12 myopes with a high myopia progression rate (< − 1.00 D/2 years), 44 myopes with a moderate progression rate (− 0.25 to − 1.00 D/2 years), and 25 myopes with no progression or a low progression rate (− 0.25 D/2 years)	Reduced P1 amplitude within the central 5 degrees (R1) in the children with high myopia progression
Chen et al. [146]	10 emmetropes (± 0.75 D) and 18 myopes (− 0.75 to − 9.50 D) with 9 stable and 9 progressive myopes (≤ − 0.50 D/2 years)	Reduced P1, N2 amplitudes and P1 implicit time within the paracentral retina (R2) in myopes. AL contributed to 17% of the variance in mfERG responses
Chen et al. [150]	11 emmetropes (± 0.75 D) and 18 myopes (− 0.75 to -9.50 D) with 9 stable and 9 progressive myopes (≤ − 0.50 D/2 years)	Reduced OPs' IT in progressive myopes
Chen et al. [145]	10 emmetropes (± 0.75 D) and 20 myopes (− 0.75 to − 9.50 D) with 10 stable and 10 progressive myopes (≤ − 0.50 D/2 years)	Delayed P1 IT in stable and progressive myopes. AL contributed to 15% of the variance in IT, while SER accounted for 27%
Wolsley et al. [160]	14 emmetropes (± 0.50 D), 14 mild myopes (− 0.75 to − 2.75 D), 14 moderate myopes (− 3.00 to -5.75 D), and 14 high myopes (≤ -6.00 D)	Reduced P1 amplitude and delayed P1 IT, with increased retinal eccentricity in high myopes
Ying et al. [144]	12 pathological myopes (AL ≥ 30.00 mm) and 24 pathological myopes (AL < 30.00 mm)	Reduced P1 amplitude, which was proportional to the neural retinal thickness in all quadrants and rings with increased AL
Shamshinova et al. [111]	46 myopes with moderate-to-high congenital myopia	Reduced P1 amplitude in all rings with increased degree of myopia and AL
Kader et al. [106]	40 emmetropes (± 0.25 D), 20 mild myopes (− 0.50 to − 3.00 D), 28 moderate myopes (− 3.25 to − 6.00 D), 40 high myopes (− 6.25 to − 15.00 D), and 40 pathological myopes (− 7.00 to − 22.00 D with 7 posterior staphyloma)	Reduced P1 amplitude and delayed P1 IT with increased degree of myopia, AL, and retinal eccentricity
Azad et al. [135]	222 emmetropes (± 0.50 D)	Maximum N1, P1, N2 amplitudes, and longest P1, N2 latencies at the fovea, which progressively decreased with increased retinal eccentricity
Park et al. [141]	30 mild myopes (− 0.50 to − 2.75 D), 25 moderate myopes (− 3.00 to − 5.75 D), 17 high myopes (− 6.00 to − 9.75 D), and 18 super high myopes (− 10.0 to − 15.0 D)	Reduced N1, P1 amplitudes and delayed P1 IT with increased degree of myopia and retinal eccentricity
Koh et al. [110]	32 myopes (≤ − 6.00 D)	Reduced P1 amplitude in the outer rings (R3–R5) with increased AL
Song et al. [142]	31 emmetropes (+ 0.75 to − 0.50 D; AL: 22 to 24 mm), 26 low-to-moderate myopes (− 0.50 to − 6.00 D; AL: 24 to 26 mm), 34 high myopes (− 6.00 to − 10.00 D; AL: 26 to 28 mm), 22 super high myopes (< − 10.00 D; AL: > 28 mm)	Reduced P1 amplitude, P1 amplitude density, and delayed P1 IT with increased degree of myopia, AL, and retinal eccentricity
Sachidanandam et al. [109]	100 eyes with axial length ranging from 21.79 to 30.55 mm and SER ranging from + 0.50 to − 18.00 D	Reduced N1, P1 amplitudes and minimal delayed corresponding IT with increased AL
Ismael et al. [140]	20 emmetropes (± 0.50 D), 20 mild myopes (− 0.50 to − 3.00 D), 20 moderate myopes (− 3.00 to − 6.00 D), and 20 high myopes (< − 6.00 D)	Reduced P1 amplitude, delayed P1 latency in all rings as well as reduced N1, P1 amplitudes, delayed N1 latency in all quadrants with increased degree of myopia, AL, retinal eccentricity, and RNFL thinning
El-Gamal et al. [143]	30 emmetropes (± 0.25 D) and 30 high myopes (≤ − 5.00 D and AL > 26 mm)	Reduced N1, P1 amplitudes and corresponding IT at almost all rings and quadrants in high myopes, which was proportional to increased AL. P1 responses were more affected
Nebbioso et al. [162]	24 emmetropes, 24 high myopes (< − 8.00 D and AL > 26 mm) with MF, and 24 high myopes without MF	Reduced P1 amplitude and delayed P1 IT with increased macular thickness in high myopes with MF

*AL* axial length, *ERG* electroretinogram, *IT* implicit time, *mfERG* multifocal electroretinogram, *MF* myopic foveoschisis, *OPs* oscillatory potentials, *R1–R5* ring 1 to ring 5 from center to periphery, RNFL retinal nerve fiber layer, *SER* spherical equivalent refractive error

Previous studies have found that the mfERG amplitude density are maximum, and the latencies of P1 and N2 waves are the longest at the central R1 (fovea), which progressively decreased with the increase in retinal eccentricity [135, 138, 142, 148]. However, P1 amplitude density reduction in case of children with progressive myopia (< − 1.00D/2 years) was reported to be significantly smaller than stable myopes at central 5 degrees (R1) [149]. Progressive myopes also showed significantly shorter implicit times for OPs arising from inner retina compared to emmetropes and stable myopes, with similar implicit times for stable myopes and emmetropes [150]. These findings collectively indicate that progression of myopia may lead to inner retinal changes and alter the electrophysiological responses at both center and periphery of the retina [150].

There are various other factors such as optical, electrical, and retinal factors that contribute to the reduced mfERG responses. For optical factors, the degraded mfERG responses are associated with the decreased retinal image size and retinal illuminance due to elongated axial length in myopes. Electrical factors such as increased electrical path and ocular resistance from the electrical sources (at retinal plane) and the ERG electrodes [151] and lower retinal cell responsivity are proposed to be linked with reduced mfERG responses in myopes [152]. Retinal factors attributing to the reduced mfERG responses in myopes are increased sub-retinal space and morphological alterations in retinal cells due to axial elongation [153, 154]. Morphological changes include decreased retinal thickness, decreased retinal photoreceptor density [155], structural changes in photoreceptor outer segment [19], and photoreceptor dysfunction [156]. The prolonged implicit times/delayed latency may also be due to altered synaptic transmission from retinal photoreceptors (primarily rods) to ON and OFF pathways [157].

### Influence of axial length on mfERG responses

Similar to ffERG, the mfERG amplitude density also shows a better correlation with axial length compared to refractive error [109, 110], which is the most important determinant of myopic mfERG responses across all five rings [140]. For every 1-mm increase in axial length, the mfERG at R1 (fovea) showed a reduction of 2.4 nV/deg^2^ and 7.4 nV/deg^2^ in N1 and P1 amplitude density, respectively, in an absence of a myopic retinal degeneration [109]. High myopes with longer axial length had decreased mfERG responses across central, paracentral [158], and outer rings (R3–R5) [110]. The central retina shows higher rates of reduction in both N1 and P1 amplitude density [109, 158]. The mean N1 and P1 mfERG amplitude density responses were reported to decrease by 6–10% per mm of axial length elongation [158]. Axial length accounted for 15% of the total variance in implicit time, while refractive error accounted for 27% [145].

Given that the mfERG responses primarily arise from retinal bipolar cells and nuclear layer, any physical or mechanical alterations to these cells will have an impact on mfERG responses [147]. Hood et al. indicated that an extensive global loss of bipolar cells essentially abolishes the overall mfERG response, whereas a localized loss exhibits a selective and localized reduction in the mfERG amplitudes with a mild-to-moderate increase in the implicit time and delayed latency [147].

### Influence of myopic retinal changes on mfERG responses

The mfERG responses gradually decline toward the periphery [159] and correlate with peripheral retinal thinning in moderate and high myopes [135, 141, 144, 160, 161]. Retinal thinning in moderate and high myopes, due to reduced middle to inner retinal (MIR) layer (from outer plexiform layer to retinal nerve fiber layer (RNFL)) thickness, was correlated with decreased spatial resolution, reduced P1 amplitude density, and delayed latency in the retinal periphery [160]. Significant correlations were observed between MIR thickness and N1, P1 amplitude density, as well as N1, P1 implicit time in the perifoveal retina corresponding to R4 [141]. However, no such significance was noted between central R1 parameters and central macular thickness [135]. The P1 amplitude density was also significantly correlated with the mean RNFL and outer macular thickness in R2, R4, R5, and R2 to R5, respectively [110].

Previous studies have shown an increase in the central subfield macular thickness (CST) [142] and a reduction in neural retinal thickness [144] with an increasing degree of myopia. The changes in CST were negatively associated with P1 amplitude [142, 162], P1 amplitude density [142], and microperimetry (MP-1) sensitivity [162]. In addition, the P1 amplitude density was found to decrease in all quadrants and rings with an increasing axial length [144]. The increase in CST in high myopes also led to an increase in P1 implicit time [142]. This shows that retinal morphological changes are closely associated with the retinal functional changes in high myopia [142, 144, 162]. Hence, the correlation between structural and functional changes is crucial for interpreting retinal health in myopes, especially in high myopia [142, 144, 161, 162].

## Pattern electroretinogram (PERG) and its responses in myopia

The PERG is a contrast-based response, driven by macular photoreceptors and originating from retinal ganglion cells [94]. It is a measure of both central retinal function and retinal ganglion cell function [163]. In macaque monkeys, both ON and OFF pathways equally contribute to the transient PERG amplitudes [164]. Clinically, transient PERG response has two main components: P50 (positive peak at 50 ms from stimulus onset) is an inner retinal component driven by macular photoreceptors and N95 (negative peak at 95 ms from stimulus onset) is the second component which is contrast-related and is generated by the retinal ganglion cells [96].

The P50 and N95 wave amplitudes of the transient PERG response were reduced in high myopes with longer axial length compared to that of emmetropes and low myopes [101, 165–167]. The amount of loss in P50 amplitude was proportional to the degree of myopia, i.e., 8% in low myopes (− 1.00 to − 3.00 D), 16% in moderate myopes (− 3.25 to − 6.00 D), and 36% in high myopes (− 6.25 to − 10.00 D), when compared with emmetropes or myopes up to − 0.75 D [166]. Similarly, the amount of loss in N95 amplitude was also proportional to the degree of myopia, i.e., 7% in low myopes, 21% in moderate myopes, and 43% in high myopes, when compared with emmetropes [166]. Although P50 wave latencies show no difference, N95 wave latencies were reported to significantly increase in high myopia [166]. The reduced P50 and N95 amplitudes in higher degrees of myopia may indicate early macular and ganglion cell dysfunction even in eyes with normal vision and a healthy appearance of the macula [165–167].

## Global-flash multifocal electroretinogram (gmfERG) and its responses in myopia

A further refinement of mfERG is the gmfERG, in which a successive insertion of a full-field or global-flash stimulus between consecutive focal flashes of a standard mfERG stimulus enhances the adaptive response, isolating the outer and inner retinal responses into two major components [168, 169]. The direct component (DC) is predominantly derived from the outer retinal cells (photoreceptors and bipolar cells), whereas the induced component (IC) is derived from the inner retinal cells (ganglion and amacrine cells) [168, 170–172].

The results from several studies that investigated gmfERG in myopia are given in Table 3. Evaluation of neural response from outer to inner retina in emmetropes and myopes using the gmfERG showed that both the DC and IC responses gradually decreased from R1 to R5. The IC responses were more affected as compared to the DC responses, indicating that the inner retina was greatly affected in myopes [173]. Both the DC and IC amplitude densities were significantly correlated for retinal mid-peripheral regions corresponding to R2 to R3 in myopic refractive error [173]. It is hypothesized that these gmfERG responses are mediated by light-adapted changes in the retinal dopaminergic system.

**Table 3:** Summary of studies on global-flash mfERG (gmfERG) and myopia

References	Participants	Outcomes
Chen et al. [173]	10 emmetropes (± 0.75 D) and 14 myopes (< − 0.75 D)	Increased DC, IC amplitudes in the paracentral retina (R2 to R3) with increased degree of myopia
Ho et al. [175]	54 myopes (SER: 0.00 to − 8.13 D)	Reduced paracentral DC amplitude for the 29% and 49% contrasts in myopes. Reduced paracentral and peripheral IC amplitudes at all contrasts measured and for the 49% contrast, respectively, in myopes. SER contributed to about 14% and 16% of the variance in DC and IC amplitude, respectively
Ho et al. [177]	22 myopic children (mean age: 11 ± 1 years)	Delayed DC (R3) and IC (R2 to R5) IT at 49% contrast in children with myopia progression. Delayed IC IT (R1) at 96% contrast in children with myopia progression
Ho et al. [176]	26 myopic children (9–13 years) with varying degrees of myopia	Reduced central DC, IC amplitudes, and paracentral IT at 49% contrast in children with myopia progression
Ho et al. [174]	52 children (9–14 years) and 19 young adults (21–28 years) with SER ranging from 0.00 to − 5.50 D	Reduced central DC amplitude at 96% contrast in myopic children. Reduced paracentral IC amplitude at 49% contrast in myopic adults
Chin et al. [179]	23 emmetropes to low myopes (+ 1.00 to − 3.25 D)	Reduced DC amplitude at a low SF, which increased with increasing SF, and decreased with increasing eccentricity
Increased IC amplitude at all SF, which decreased with increasing eccentricity		
Li et al. [178]	56 emmetropic children (± 0.50 D)	Reduced central IC amplitudes at 49% contrast with the myopic changes in SER and AL after 1 year

*AL* axial length, *DC* direct component, *ERG* electroretinogram, *gmfERG* global-flash multi-focal electroretinogram, *IC* induced component, *IT* implicit time, *R1–R5* ring 1 to ring 5 from center to periphery, *SER* spherical equivalent refractive error, *SF* spatial frequency

The gmfERG on myopic children with different contrast levels exhibited a significant reduction in central macular (R1) DC amplitude density at 96% contrast, while the IC amplitude density was unaffected [174]. But myopic adults showed a significant reduction in the paracentral DC amplitude density for 29% and 49% contrasts [175]. The IC amplitude density in myopic adults is reduced for all measured contrast levels in both central and peripheral retinas [175]. There were no significant changes for both DC and IC implicit times in children and adults [174, 175]. Overall, these findings suggest that gmfERG-derived inner and outer retinal function in myopes vary significantly with age and retinal eccentricity.

A similar contrast-based gmfERG setup was used to determine whether myopia progression measured over 1 year was associated with changes in retinal function. At 49% contrast, both the DC and IC amplitude densities at the macula (central R1) were significantly reduced with the progression of myopia [176–178]. The DC and IC implicit times were also reduced considerably in the paracentral retinal region [176, 177]. However, the high-contrast responses remained unaffected by the myopia progression [176–178]. The findings indicate that myopia progression in children alters the inner retinal function at central retina, along with partial involvement of paracentral retina [176, 177]. The retinal electrophysiological functions seem not only differentially affected in children and adult myopes, but also in outer and inner retina that differentially process the spatial details [179].

## Electroretinogram responses to anti-myopia strategies

Given an alarming rise in the prevalence of myopia worldwide, various optical, pharmacological, and environmental strategies are being incorporated to prevent the development of myopia [180] as well as to slow down the rate of myopia progression in children [181, 182]. One of the popular optical anti-myopia strategies includes orthokeratology [183, 184], which decreases the hyperopic defocus at the peripheral retina in myopic eyes [185–187]. A recent investigation on the effect of 60 days of orthokeratology treatment on PERG reported significantly delayed implicit time of P50 and N95 wave, but no effect on PERG amplitudes [188]. Because the blur induced by orthokeratology and other peripheral defocus inducing anti-myopia strategies is not the same across all retinal eccentricities, it would be useful to use the mfERG to investigate how ERG responses vary in different retinal regions.

Besides optical anti-myopia strategies, pharmacological management of myopia progression with atropine eye drops has also been one of the most effective strategies to control myopia progression in children [181, 189–193]. The majority of previous ERG studies that investigated different concentrations of atropine eye drops (0.01%, 0.1%, 0.5%, and 1%) on retinal signals reported no significant effect of atropine on retinal function as demonstrated by ffERG [194–196], mfERG [194, 197], or PERG [198] in young myopic children (< 14 years of age). However, there are a few studies that report contradictory results. Firstly, Khanal et al. [199] reported that 0.1% atropine eye drops resulted in a 14% reduction of dark-adapted 3.0 OP amplitudes and 4% delay in the a-wave implicit time of dark-adapted 10.0 ERG (stronger ffERG), indicating that atropine could alter neural activity in inner retina and activity of photoreceptors, respectively [199]. Secondly, Kothari et al. [194] reported a reduction in the P50 amplitude of PERG with 0.01% atropine eye drops, indicating that the induced optical blur due to cycloplegia and mydriasis may alter signal transmission in inner retina (amacrine cells) [194]. Lastly, it was reported that gmfERG responses increased with 0.1% atropine eye drops in the presence of optically induced myopic defocus, suggesting that the atropine may enhance the effects of myopic defocus in the inner layers of the peripheral retina in controlling the eye growth for potential anti-myopia effects [200]. Overall, the literature related to the influence of atropine eye drops on altering retinal signals and regulating ocular growth is sparse and warrants further in-depth investigations to improve understanding of this important relationship and mechanism.

## Conclusions

To summarize, there are significant changes in retinal function, as assessed from ERG testing in myopes, and these changes strongly correlate with axial length and the degree of myopia. Although some investigations with the ffERG show significantly reduced dark-adapted and light-adapted a- and b-wave amplitudes with increasing degree of myopic refractive error, there is some evidence that dark-adapted responses are further attenuated than the light-adapted responses. These findings suggest that myopia may be associated with reduced photoreceptor (mainly rod response) and ON-bipolar cell activity. Several studies with the mfERG show reduced P1 amplitude density in myopes, suggesting alterations in retinal ON-and-OFF cone bipolar cells in myopia. The mfERG amplitude density associated with refractive error varies significantly with retinal eccentricity (larger reduction in peripheral retina than in the fovea), axial length, and the degree of myopia. Finally, studies have reported reduced PERG amplitudes and gmfERG amplitude density in both the central and paracentral retina in high myopia. While these studies illustrate important associations between myopic refractive error and changes in retinal electrical activity, there has been limited work to understand the longitudinal changes in the ERG and how they relate to myopia progression in younger eyes. Future work investigating electrophysiological responses in combination with the measurements of retinal structural changes (using optical coherence tomography) will provide valuable insights into how retinal electrical changes may influence ocular growth and refractive error development in humans. Given the availability and wide use of optical and pharmacological anti-myopia management strategies that are known to act at retinal level (such as orthokeratology, multifocal contact lenses, and atropine), it would also be interesting for future studies to examine how these anti-myopia interventions interact with retinal signals to prevent myopia.
