# Correction to: Electroretinogram responses in myopia: a review

**DOI:** 10.1007/s10633-022-09876-w

**Published:** 2022-08-18

**Authors:** Satish Kumar Gupta, Ranjay Chakraborty, Pavan Kumar Verkicharla

**Affiliations:** 1 Myopia Research Lab, Prof. Brien Holden Eye Research Centre, Brien Holden Institute of Optometry and Vision Sciences, Kallam Anji Reddy Campus, L V Prasad Eye Institute, Hyderabad, India; 2 Caring Futures Institute, College of Nursing and Health Sciences, Optometry and Vision Science, Flinders University, Adelaide, South Australia Australia

## Correction to: Doc Ophthalmol 10.1007/s10633-021-09857-5

In the published article, there are few changes requested by the author. The changes are as follows:

In section “Multifocal electroretinogram (mfERG) and its responses in myopia”—last sentence of 2nd paragraph should read as:

“In humans, the N1 component primarily originates from the ON- and OFF-cone bipolar cells with minimal contribution from the cone photoreceptors.”

In addition, the following figure captions and table footntoes should read as follows: 

Figure 2: Panel A: The b-wave amplitudes plotted against the moderate (MM) and high myopia (HM) are those associated with moderate and advanced chorioretinal degeneration, respectively, within the group of pathological myopes (PM) studied [119]. Panel A and B: These older studies used non-standard stimuli, strong flashes in dark-adapted participants, and such amplitudes are more comparable to those in panels G and H dark-adapted ‘combined’ ERGs. Panel D: Should be labeled “Combined ERG” as Wan et al [122] used the ISCEV Standard dark-adapted 3.0 stimulus. A revised figure (2S) with corrected labels is available in the electronic supplemental material.

Figure 4: Panels E, H, and I: The y-axis in these panels is microvolt (µV), not conventional density units (nV/deg^2^). (The angular size of the stimulus elements for conversion to density are not available.)

Table 1: Several studies in this table used non-standard ERG protocols or extended protocols [119, 112, 113, 122]; The study by Ishikawa et al. [120] used a focal macular ERG protocol

Tables 1–3: Some details of the stimuli and participants are omitted from tables. Tables 1S, 2S and 3S in the electronic supplement include the omitted information to allow clearer comparisons between studies.

## Supplementary Information

Below is the link to the electronic supplementary material.

Supplementary file 1 (DOCX 1014 kb)

Supplementary file 2 (PDF 723 kb)

