# Supplementary material for: Correction to: Electroretinogram responses in myopia: a review
Source: Doc Ophthalmol. 2022 Aug 18;145(2):97–8. doi: 10.1007/s10633-022-09876-w (PMC9470685; doi:10.1007/s10633-022-09876-w)
Supplement: Supplementary file 1 — (DOCX 1014 kb) [file 10633_2022_9876_MOESM1_ESM.docx]

Electronic Supplemental material for Gupta et al DOI 10.1007/s10633-021-09857-5

**Table 1S** **Summary of studies using flash and focal macular ERG in myopia** (with supplemental participant and stimulus information)

| **References** | **Participants and *stimuli** | **Outcomes** |
| --- | --- | --- |
| Blach et al. [[119](#_ENREF_119)] | 25 emmetropes and 30 high myopes (- 9.00 to - 24.00 D) with degenerative fundus changes  *Dark-adapted strong flash (non-standard) | Increased a-wave and reduced b-wave amplitude with increased degree of chorioretinal degeneration in high myopia |
| Malik et al. [[112](#_ENREF_112)] | 43 myopes with degenerative fundus changes and 37 myopes with normal fundus  *Light-adapted strong flash (non-standard) | Reduced a- and b-wave amplitudes as the degree of myopia increased.  Decreased ffERG amplitudes in eyes with degenerative fundus changes, irrespective of the degree of myopia |
| Perlman et al. [[107](#_ENREF_107)] | 31 high hypermetropes (> + 5.00 D), 7 high myopes (< - 6.00 D), and 7 unilateral or bilateral aphakics  *Light-adapted strong flash and dark-adapted luminance series | Reduced scotopic a- and b-wave and photopic b-wave amplitudes in high myopes |
| Ishikawa et al. [[120](#_ENREF_120)] | 66 high myopes and 76 emmetropes  *Focal macular ERG | Reduced a- and b-wave amplitudes in high myopes with only tigroid fundus.  Reduced a- and b-wave, and OPs' amplitudes and increased implicit times in posterior staphyloma involving the macula |
| Westall et al. [[108](#_ENREF_108)] | 33 high myopes (- 6.00 to - 14.50 D), 8 mild myopes (- 3.00 to - 5.00 D), and 19 small SER (+ 0.75 to - 2.75 D)  *Light- and dark-adapted ERGs | Reduced rod-cone a- and b-wave, cone b-wave, and dark-adapted OPs' amplitudes in high myopes, which was proportional to increased AL |
| Yoshii et al. [[113](#_ENREF_113)] | 14 emmetropes (- 0.50 to - 3.50 D) and 16 high myopes (- 7.00 to - 11.50 D)  *40-degree light-adapted single and double  flash ERGs | Reduced nonlinear component of the ERG amplitudes from the posterior pole of the fundus in high myopes |
| Flitcroft et al. [[101](#_ENREF_101)] | 15 high myopes (≤ - 6.00 D), 19 low myopes (- 0.75 to - 6.00 D), 35 emmetropes (- 0.75 to + 1.50 D), 44 low hyperopes (+ 1.50 to + 6.00 D), and 10 high hyperopes (≥ + 6.00 D) referred for reduced vision or  nystagmus | Higher rates of abnormal ffERG in patients with high ammetropia |
| Shamshinova et al. [[111](#_ENREF_111)] | 46 myopes with moderate-to-high congenital myopia | Reduced b-wave amplitude with increased degree of myopia and AL |
| Kader et al. [[106](#_ENREF_106)] | 40 emmetropes (± 0.25 D), 20 mild myopes (- 0.50 to - 3.00 D), 28 moderate myopes (- 3.25 to - 6.00 D), 40 high myopes (- 6.25 to - 15.00 D), and 40 pathological myopes (- 7.00 to - 22.00 D with 7 posterior staphyloma) | Reduced scotopic, photopic, and combined b-wave, OPs', and 30 Hz flicker amplitudes as well as delayed latencies in high myopes, which was proportional to increased AL |
| Wang et al. [[117](#_ENREF_117)] | 64 early-onset high myopes and 20 late-onset high myopes | Reduced scotopic b-wave, photopic a- and b- wave, and combined a- and b- wave amplitudes in early-onset high myopes |
| Koh et al. [[110](#_ENREF_110)] | 32 myopes (≤ - 6.00 D) | Reduced scotopic b-wave, photopic a- and b- wave, and 30 Hz flicker b-wave amplitudes with increased degree of myopia and AL |
| Sachidanandam et al. [[109](#_ENREF_109)] | 100 eyes with AL ranging from 21.79 to 30.55 mm and SER ranging from + 0.50 to - 18.00 D | Reduced both scotopic and photopic a- and b-wave amplitudes and minimal delayed corresponding IT with increased AL |
| Wan et al. [[122](#_ENREF_122)] | 19 emmetropes (± 0.25 D), 18 low myopes (- 0.50 to - 3.00 D), 23 moderate myopes (- 3.25 to - 6.00 D), and 16 high myopes (≤ - 6.25 D)  *ISCEV Standard ERGs with power spectrum analysis of OPs | Increased combined a- and b-wave amplitudes and OPs' peak frequency with increased degree of myopia |

*AL* axial length, *ERG* electroretinogram, *ffERG* full-field flash electroretinogram, *IT* implicit time, *OPs'* oscillatory potentials, *SER* spherical equivalent refractive error

*ISCEV Standard full-field stimuli are used except as otherwise specified

**Table 2S:** Summary of studies using multi-focal ERG (mfERG) in myopia

| **References** | **Participants** | **Outcomes** |
| --- | --- | --- |
| Kawabata et al. [[138](#_ENREF_138)] | 10 emmetropes/low myopes (+ 1.00 to - 3.00 D), 10 moderate myopes (- 3.25 to - 6.00 D), 10 high myopes (≤ - 6.25 D) | Reduced N1, P1 amplitudes and delayed corresponding latencies with increased degree of myopia and retinal eccentricity |
| Sun et al. [[148](#_ENREF_148)] | 20 emmetropes, 20 mild myopes, 20 moderate myopes, and 20 high myopes | Reduced N1, P1, N2 amplitudes and corresponding response density with increased degree of myopia and retinal eccentricity |
| Chan et al. [[158](#_ENREF_158)] | 30 subjects with AL ranging from 23.72 to 28.13 mm and SER ranging from 0.00 to -10.50 D | Reduced P1 amplitude in the central (R1) and reduced N1, P1 amplitudes in the paracentral region (R3) with increased degree of myopia and AL.  The mfERG amplitude reduced by about 6–10% per 1-mm increase in AL. |
| Luu et al. [[139](#_ENREF_139)] | 104 children and 31 adults with SER ranging from 0.00 to -10.00 D | Reduced N1, P1, N2 amplitudes and delayed corresponding IT with increased degree of myopia in adults |
| Luu et al. [[149](#_ENREF_149)] | 12 myopes with a high myopia progression rate (< - 1.00 D/2 years), 44 myopes with a moderate progression rate (- 0.25 to - 1.00 D/2 years), and 25 myopes with no progression or a low progression rate (- 0.25 D/2 years) | Reduced P1 amplitude within the central 5 degrees (R1) in children with high myopia progression |
| Chen et al. [[146](#_ENREF_146)] | 10 emmetropes (± 0.75 D) and 18 myopes (- 0.75 to - 9.50 D) with 9 stable and 9 progressive myopes (≤ - 0.50 D/2 years) | Reduced P1, N2 amplitudes and P1 implicit time within the paracentral retina (R2) in myopes.  AL contributed to 17% of the variance in mfERG responses |
| Chen et al. [[150](#_ENREF_150)] | 11 emmetropes (± 0.75 D) and 18 myopes (- 0.75 to - 9.50 D) with 9 stable and 9 progressive myopes (≤ - 0.50 D/2 years) | Reduced OPs' IT in progressive myopes |
| Chen et al. [[145](#_ENREF_145)] | 10 emmetropes (± 0.75 D) and 20 myopes (- 0.75 to - 9.50 D) with 10 stable and 10 progressive myopes (≤ - 0.50 D/2 years) | Delayed P1 IT in stable and progressive myopes. AL contributed to 15% of the variance in IT, while SER accounted for 27% |
| Wolsley et al. [[160](#_ENREF_160)] | 14 emmetropes (± 0.50 D), 14 mild myopes (- 0.75 to - 2.75 D), 14 moderate myopes (- 3.00 to - 5.75 D), and 14 high myopes (≤ - 6.00 D) | Reduced P1 amplitude and delayed P1 IT, with increased retinal eccentricity in high myopes |
| Ying et al. [[144](#_ENREF_144)] | 12 pathological myopes (AL ≥ 30.00 mm) and 24 pathological myopes (AL < 30.00 mm) | Reduced P1 amplitude, which was proportional to the neural retinal thickness in all quadrants and rings with increased AL |
| Shamshinova et al. [[111](#_ENREF_111)] | 46 myopes with moderate-to-high congenital myopia | Reduced P1 amplitude in all rings with increased degree of myopia and AL |
| Kader et al. [[106](#_ENREF_106)] | 40 emmetropes (± 0.25 D), 20 mild myopes (- 0.50 to - 3.00 D), 28 moderate myopes (- 3.25 to - 6.00 D), 40 high myopes (- 6.25 to - 15.00 D), and 40 pathological myopes (- 7.00 to - 22.00 D with 7 posterior staphyloma) | Reduced P1 amplitude and delayed P1 IT with increased degree of myopia, AL, and retinal eccentricity |
| Azad et al. [[135](#_ENREF_135)] | 222 emmetropes (± 0.50 D) | Maximum N1, P1, N2 amplitudes, and longest P1, N2 latencies at the fovea, which progressively decreased with increased retinal eccentricity |
| Park et al. [[141](#_ENREF_141)] | 30 mild myopes (- 0.50 to - 2.75 D), 25 moderate myopes (- 3.00 to - 5.75 D), 17 high myopes (- 6.00 to - 9.75 D), and 18 super high myopes (- 10.0 to - 15.0 D) | Reduced N1, P1 amplitudes and delayed P1 IT with increased degree of myopia and retinal eccentricity |
| Koh et al. [[110](#_ENREF_110)] | 32 myopes (≤ - 6.00 D) | Reduced P1 amplitude in the outer rings (R3-R5) with increased AL |
| Song et al. [[142](#_ENREF_142)] | 31 emmetropes (+ 0.75 to - 0.50 D; AL: 22 to 24 mm), 26 low-to-moderate myopes (- 0.50 to - 6.00 D; AL: 24 to 26 mm), 34 high myopes (- 6.00 to -10.00 D; AL: 26 to 28 mm), 22 super high myopes (< - 10.00 D; AL: > 28 mm) | Reduced P1 amplitude, P1 amplitude density, and delayed P1 IT with increased degree of myopia, AL, and retinal eccentricity |
| Sachidanandam et al. [[109](#_ENREF_109)] | 100 eyes with AL ranging from 21.79 to 30.55 mm and SER ranging from + 0.50 to - 18.00 D | Reduced N1, P1 amplitudes and minimal delayed corresponding IT with increased AL |
| Ismael et al. [[140](#_ENREF_140)] | 20 emmetropes (± 0.50 D), 20 mild myopes (- 0.50 to - 3.00 D), 20 moderate myopes (- 3.00 to -6.00 D), and 20 high myopes (< - 6.00 D) | Reduced P1 amplitude, delayed P1 latency in all rings as well as reduced N1, P1 amplitudes, delayed N1 latency in all quadrants with increased degree of myopia, AL, retinal eccentricity, and RNFL thinning |
| El-Gamal et al. [[143](#_ENREF_143)] | 30 emmetropes (± 0.25 D) and 30 high myopes (≤ - 5.00 D and AL > 26 mm) | Reduced N1, P1 amplitudes and corresponding IT at almost all rings and quadrants in high myopes, which was proportional to increased AL.  P1 responses were more affected |
| Nebbioso et al. [[162](#_ENREF_162)] | 24 emmetropes, 24 high myopes (< - 8.00 D and AL > 26 mm) with MF, and 24 high myopes without MF | Reduced P1 amplitude and delayed P1 IT with increased macular thickness in high myopes with MF |

*AL* axial length, *ERG* electroretinogram, *IT* implicit time, *mfERG* multi-focal electroretinogram, *MF* myopic foveoschisis, *OPs'* oscillatory potentials, *R1-R5* ring 1 to ring 5 from center to periphery, RNFL retinal nerve fiber layer, *SER* spherical equivalent refractive error

**Table 3S** Summary of studies using global-flash mfERG (gmfERG) in myopia

| **References** | **Participants** | **Outcomes** |
| --- | --- | --- |
| Chen et al. [[173](#_ENREF_173)] | 10 emmetropes (± 0.75 D) and 14 myopes (< - 0.75 D) | Increased DC, IC amplitudes in the paracentral retina (R2 to R3) with increased degree of myopia |
| Ho et al. [[175](#_ENREF_175)] | 54 myopes (SER: 0.00 to - 8.13 D) | Reduced paracentral DC amplitude for the 29% and 49% contrasts in myopes.  Reduced paracentral and peripheral IC amplitudes at all contrasts measured and for the 49% contrast, respectively, in myopes.  SER contributed to about 14% and 16% of the variance in DC and IC amplitude, respectively. |
| Ho et al. [[177](#_ENREF_177)] | 22 myopic children (mean age: 11 ± 1 years) | Delayed DC (R3) and IC (R2 to R5) IT at 49% contrast in children with myopia progression.  Delayed IC IT (R1) at 96% contrast in children with myopia progression |
| Ho et al. [[176](#_ENREF_176)] | 26 myopic children (9-13 years) with varying degrees of myopia | Reduced central DC, IC amplitudes, and paracentral IT at 49% contrast in children with myopia progression |
| Ho et al. [[174](#_ENREF_174)] | 52 children (9-14 years) and 19 young adults (21-28 years) with SER ranging from 0.00 to - 5.50 D | Reduced central DC amplitude at 96% contrast in myopic children.  Reduced paracentral IC amplitude at 49% contrast in myopic adults |
| Chin et al. [[179](#_ENREF_179)] | 23 emmetropes to low myopes (+ 1.00 to - 3.25 D) | Reduced DC amplitude at a low SF, which increased with increasing SF, and decreased with increasing eccentricity.  Increased IC amplitude at all SF, which decreased with increasing eccentricity |
| Li et al. [[178](#_ENREF_178)] | 56 emmetropic children (± 0.50 D) | Reduced central IC amplitudes at 49% contrast with the myopic changes in SER and AL after 1 year |

*AL* axial length, *DC* direct component, *ERG* electroretinogram, *gmfERG* global-flash multi-focal electroretinogram, *IC* induced component, *IT* implicit time, *R1-R5* ring 1 to ring 5 from center to periphery, *SER* spherical equivalent refractive error, *SF* spatial frequency

**Supplemental Figures:**

**Fig. 1S** Representative waveforms and parameters of **A** dark‐adapted 3.0 ERG and **C** light‐adapted 3.0 ERG of full‐field electroretinogram (ffERG). Average ffERG waveforms from **B** dark‐adapted 3.0 ERG and **D** light‐adapted 3.0 ERG from 100 eyes with axial length (AL) ranging from 21.79 to 30.55 mm and spherical equivalent refractive error (SER) ranging from + 0.50 to − 18.00 D. All participants were divided into seven different groups based on their mean AL: Group 1 (22.40 mm), Group 2 (23.10 mm), Group 3 (24.26 mm), Group 4 (25.51 mm), Group 5 (26.34 mm), Group 6 (27.5 mm), and Group 7 (29.55 mm). The two values listed for each group under latencies and values, respectively, indicate the peak latencies (Δ ms) and amplitudes (Δ µV) of a ffERG a-wave (negative values from baseline) and b-wave (positive values from the a-wave). Adapted with permission from Sachidanandam et al. (2017) [[109](#_ENREF_109)].

**Fig. 2(S)**  Mean b-wave amplitudes (µV) for scotopic (**A**), photopic (**B, C**), and combined response (**D-H**) of full-field flash ERG (ffERG) reported by each study in emmetropia (*Emm*) and various grades of myopia (*LM* low myopia, *MM* moderate myopia, *HM* high myopia, *SM* severe myopia, and *PM* pathological myopia)

**Fig. 3(S)** **A** A typical first-order kernel multifocal electroretinogram (mfERG) waveform and its parameters. **B** Average six mfERG ring responses from 100 eyes with axial length (AL) ranging from 21.79 to 30.55 mm and spherical equivalent refractive error (SER) ranging from + 0.50 to − 18.00 D. All participants were divided into seven different groups based on their mean AL: Group 1 (22.40 mm), Group 2 (23.10 mm), Group 3 (24.26 mm), Group 4 (25.51 mm), Group 5 (26.34 mm), Group 6 (27.5 mm), and Group 7 (29.55 mm). The two values listed for each group under latencies and under values, respectively, indicate the difference in latencies (Δ ms) and amplitude density (Δ nV/deg^2^) of a mfERG N1 (negative values from baseline) and P1 (positive values from N1) wave. Adapted with permission from Sachidanandam et al. (2017) [[109](#_ENREF_109)].

**Fig. 4** Mean multifocal electroretinogram (mfERG) P1 amplitude density (nV/deg^2^) at each ring (R1: filled purple circles, R2: unfilled blue circles, R3: filled green squares, R4: unfilled orange squares, R5: filled red triangles, and R6: black cross) reported by each study in emmetropia (*Emm*) and various grades of myopia (*LM* low myopia, *MM* moderate myopia, *HM* high myopia, *SM* severe myopia, and *PM* pathological myopia)

*The studies in the last row of panels (**G-I**) report the mfERG amplitudes in microvolt (µV), and not in conventional density units (nV/deg^2^). The mfERG P1 amplitudes are shown in nV (nanovolt) not nV/deg^2^.
