# Supplementary material for: Correction to: Electroretinogram responses in myopia: a review
Source: Doc Ophthalmol. 2022 Aug 18;145(2):97–8. doi: 10.1007/s10633-022-09876-w (PMC9470685; doi:10.1007/s10633-022-09876-w)
Supplement: Supplementary file 2 — (PDF 723 kb) [file 10633_2022_9876_MOESM2_ESM.pdf]

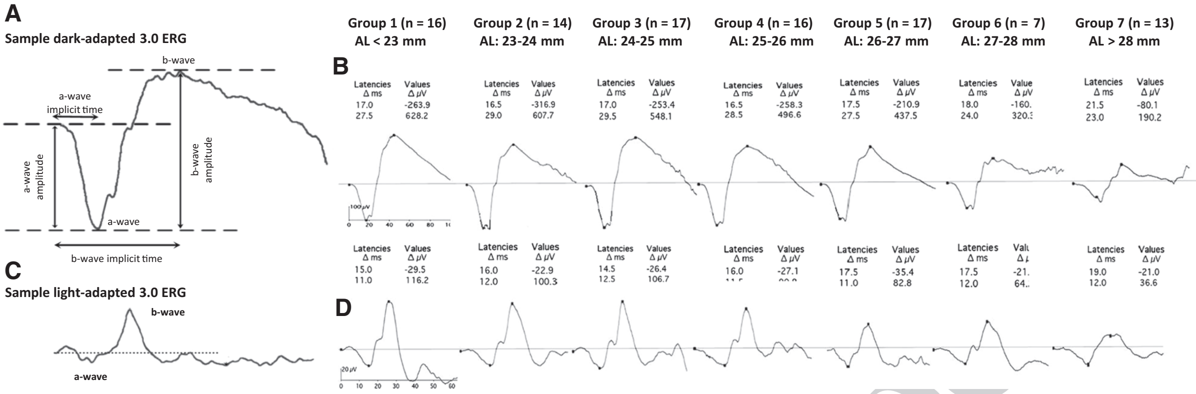

Fig. 1 Sample of normal waveforms and parameters of **A** dark-adapted 3.0 ERG and **C** light-adapted 3.0 ERG of full-field electroretinogram (ffERG). Average ffERG responses from **B** dark-adapted 3.0 ERG and **D** light-adapted 3.0 ERG from 100 eyes with axial length (AL) ranging from 21.79 to 30.55 mm and spherical equivalent refractive error (SER) ranging from + 0.50 to − 18.00 D. All participants were divided into seven different groups based on their mean AL: Group 1 (22.40 mm), Group 2 (23.10 mm), Group 3 (24.26 mm), Group 4 (25.51 mm), Group 5 (26.34 mm), Group 6 (27.5 mm), and Group 7 (29.55 mm). The two values listed for each group under latencies and values, respectively, indicate the change (Δ) in latencies (Δ ms) and amplitudes (Δ μV) of a ffERG a-wave (negative values) and b-wave (positive values). Adapted with permission from Sachidanandam et al. (2017) [109]

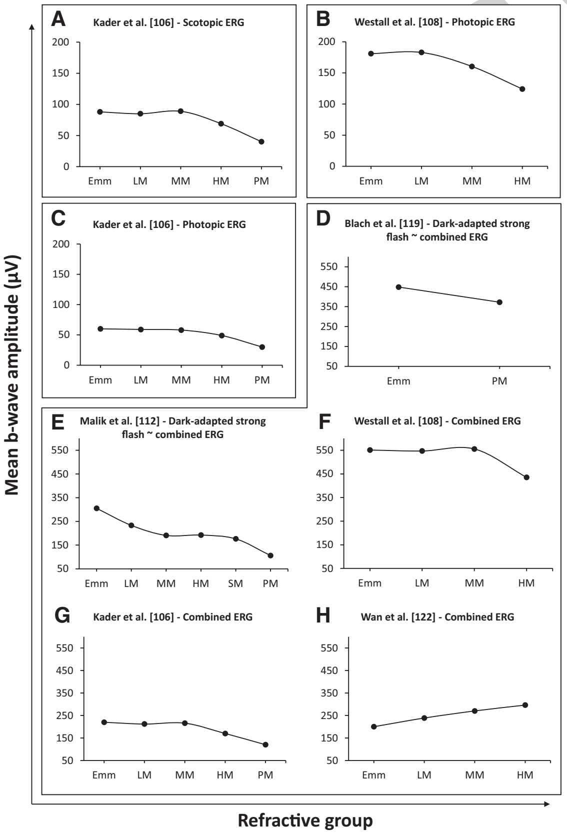

◀ **Fig. 2** **Panel A** The b-wave amplitudes plotted against the moderate (MM) and high myopia (HM) are those associated with moderate and advanced chorioretinal degeneration, respectively, within the group of pathological myopes (PM) studied [119]. **Panel A** and **B** These older studies used non-standard stimuli, strong flashes in dark-adapted participants, and such amplitudes are more comparable to those in panels G and H dark-adapted 'combined' ERGs. **Panel D** Should be labeled "Combined ERG" as Wan et al [122] used the ISCEV Standard dark-adapted 3.0 stimulus. A revised figure (2S) with corrected labels is available in the electronic supplemental material.

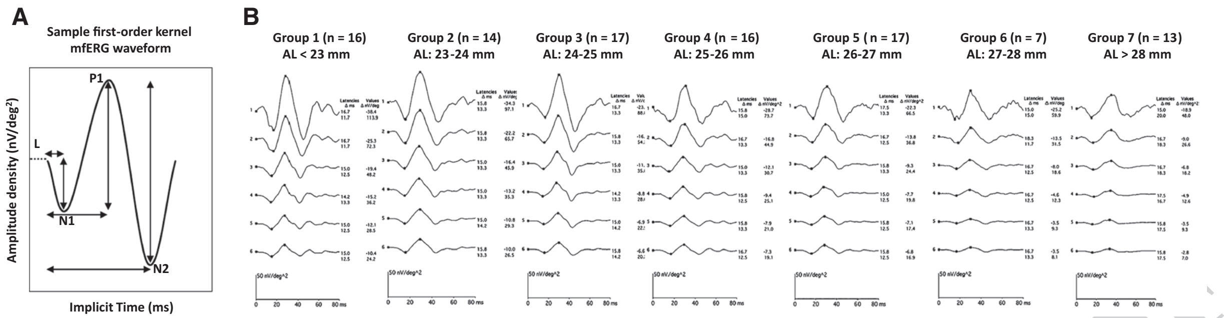

Fig. 3 **A** Sample of a normal first-order kernel multifocal electroretinogram (mfERG) waveform and its parameters. **B** Average six mfERG ring responses from 100 eyes with axial length (AL) ranging from 21.79 to 30.55 mm and spherical equivalent refractive error (SER) ranging from + 0.50 to − 18.00 D. All participants were divided into seven different groups based on their mean AL: Group 1 (22.40 mm), Group 2 (23.10 mm), Group 3 (24.26 mm), Group 4 (25.51 mm), Group 5 (26.34 mm), Group 6 (27.5 mm), and Group 7 (29.55 mm). The two values listed for each group under latencies and under values, respectively, indicate the change (Δ) in latencies (Δ ms) and amplitude density (Δ nV/deg^2) of a mfERG N1 (negative values) and P1 (positive values) wave. Adapted with permission from Sachidanandam et al. (2017) [109]

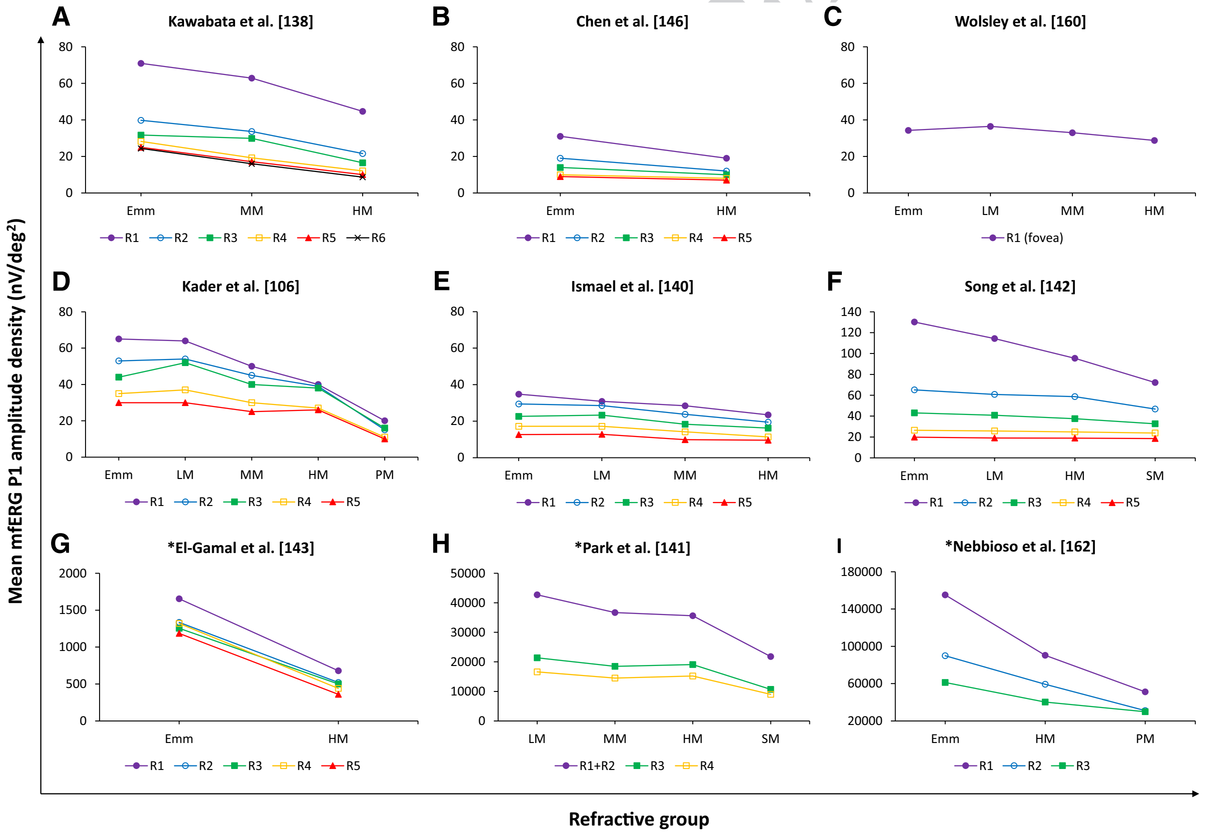

Fig. 4 **Panels E**, **H**, and **I** The y-axis in these panels is microvolt (μV), not conventional density units (nV/deg^2). (The angular size of the stimulus elements for conversion to density are not available.)

Table 1 Several studies in this table used non-standard ERG protocols or extended protocols [112, 113, 119, 122]; The study by Ishikawa et al. [120] used a focal macular ERG protocol

References	Participants and *stimuli	Outcomes
Blach et al. [119]	25 emmetropes and 30 high myopes (− 9.00 to − 24.00 D) with degenerative fundus changes<br>*Dark-adapted strong flash (non-standard)	Increased a-wave and reduced b-wave amplitude with increased degree of chorioretinal degeneration in high myopia
Malik et al. [112]	43 myopes with degenerative fundus changes and 37 myopes with normal fundus<br>*Light-adapted strong flash (non-standard)	Reduced a- and b-wave amplitudes as the degree of myopia increased<br>Decreased ffERG amplitudes in eyes with degenerative fundus changes, irrespective of the degree of myopia
Perlman et al. [107]	31 high hypermetropes (> + 5.00 D), 7 high myopes (< − 6.00 D), and 7 unilateral or bilateral aphakics<br>*Light-adapted strong flash and dark-adapted luminance series	Reduced scotopic a- and b-wave and photopic b-wave amplitudes in high myopes
Ishikawa et al. [120]	66 high myopes and 76 emmetropes<br>*Focal macular ERG	Reduced a- and b-wave amplitudes in high myopes with only tigroid fundus<br>Reduced a- and b-wave, and OPs' amplitudes and increased implicit times in posterior staphyloma involving the macula
Westall et al. [108]	33 high myopes (− 6.00 to − 14.50 D), 8 mild myopes (− 3.00 to − 5.00 D), and 19 small SER (+ 0.75 to − 2.75 D)<br>*Light- and dark-adapted ERGs	Reduced rod-cone a- and b-wave, cone b-wave, and dark-adapted OPs' amplitudes in high myopes, which was proportional to increased AL
Yoshii et al. [113]	14 emmetropes (− 0.50 to − 3.50 D) and 16 high myopes (− 7.00 to − 11.50 D)<br>*40-degree light-adapted single and double flash ERGs	Reduced nonlinear component of the ERG amplitudes from the posterior pole of the fundus in high myopes
Flitcroft et al. [101]	15 high myopes (≤ − 6.00 D), 19 low myopes (− 0.75 to − 6.00 D), 35 emmetropes (− 0.75 to + 1.50 D), 44 low hyperopes (+ 1.50 to + 6.00 D), and 10 high hyperopes (≥ + 6.00 D) referred for reduced vision or nystagmus	Higher rates of abnormal ffERG in patients with high ammetropia
Shamshinova et al. [111]	46 myopes with moderate-to-high congenital myopia	Reduced b-wave amplitude with increased degree of myopia and AL
Kader et al. [106]	40 emmetropes (± 0.25 D), 20 mild myopes (− 0.50 to − 3.00 D), 28 moderate myopes (− 3.25 to − 6.00 D), 40 high myopes (− 6.25 to − 15.00 D), and 40 pathological myopes (− 7.00 to − 22.00 D with 7 posterior staphyloma)	Reduced scotopic, photopic, and combined b-wave, OPs', and 30 Hz flicker amplitudes as well as delayed latencies in high myopes, which was proportional to increased AL
Wang et al. [117]	64 early-onset high myopes and 20 late-onset high myopes	Reduced scotopic b-wave, photopic a- and b- wave, and combined a- and b- wave amplitudes in early-onset high myopes
Koh et al. [110]	32 myopes (≤ − 6.00 D)	Reduced scotopic b-wave, photopic a- and b- wave, and 30 Hz flicker b-wave amplitudes with increased degree of myopia and AL
Sachidanandam et al. [109]	100 eyes with AL ranging from 21.79 to 30.55 mm and SER ranging from + 0.50 to − 18.00 D	Reduced both scotopic and photopic a- and b-wave amplitudes and minimal delayed corresponding IT with increased AL
Wan et al. [122]	19 emmetropes (± 0.25 D), 18 low myopes (− 0.50 to − 3.00 D), 23 moderate myopes (− 3.25 to − 6.00 D), and 16 high myopes (≤ − 6.25 D)<br>*ISCEV Standard ERGs with power spectrum analysis of OPs	Increased combined a- and b-wave amplitudes and OPs' peak frequency with increased degree of myopia

AL axial length, *ERG* electroretinogram, *ffERG* full-field flash electroretinogram, *IT* implicit time, *OPs'* oscillatory potentials, *SER* spherical equivalent refractive error

*ISCEV Standard full-field stimuli are used except as otherwise specified

UNCORRECTED PROOF

Journal : Medium 10633 | Article No : 9876 | Pages : 7 | MS Code : 9876 | Dispatch : 8-7-2022

Table 2 Summary of studies using multi-focal ERG (mfERG) in myopia

References	Participants	Outcomes
Kawabata et al. [138]	10 emmetropes/low myopes (+ 1.00 to − 3.00 D), 10 moderate myopes (− 3.25 to − 6.00 D), 10 high myopes (≤ − 6.25 D)	Reduced N1, P1 amplitudes and delayed corresponding latencies with increased degree of myopia and retinal eccentricity
Sun et al. [148]	20 emmetropes, 20 mild myopes, 20 moderate myopes, and 20 high myopes	Reduced N1, P1, N2 amplitudes and corresponding response density with increased degree of myopia and retinal eccentricity
Chan et al. [158]	30 subjects with AL ranging from 23.72 to 28.13 mm and SER ranging from 0.00 to − 10.50 D	Reduced P1 amplitude in the central (R1) and reduced N1, P1 amplitudes in the paracentral region (R3) with increased degree of myopia and AL<br>The mfERG amplitude reduced by about 6–10% per 1-mm increase in AL
Luu et al. [139]	104 children and 31 adults with SER ranging from 0.00 to − 10.00 D	Reduced N1, P1, N2 amplitudes and delayed corresponding IT with increased degree of myopia in adults
Luu et al. [149]	12 myopes with a high myopia progression rate (< − 1.00 D/2 years), 44 myopes with a moderate progression rate (− 0.25 to − 1.00 D/2 years), and 25 myopes with no progression or a low progression rate (− 0.25 D/2 years)	Reduced P1 amplitude within the central 5 degrees (R1) in children with high myopia progression
Chen et al. [146]	10 emmetropes (± 0.75 D) and 18 myopes (− 0.75 to − 9.50 D) with 9 stable and 9 progressive myopes (≤ − 0.50 D/2 years)	Reduced P1, N2 amplitudes and P1 implicit time within the paracentral retina (R2) in myopes<br>AL contributed to 17% of the variance in mfERG responses
Chen et al. [150]	11 emmetropes (± 0.75 D) and 18 myopes (− 0.75 to − 9.50 D) with 9 stable and 9 progressive myopes (≤ − 0.50 D/2 years)	Reduced OPs' IT in progressive myopes
Chen et al. [145]	10 emmetropes (± 0.75 D) and 20 myopes (− 0.75 to − 9.50 D) with 10 stable and 10 progressive myopes (≤ − 0.50 D/2 years)	Delayed P1 IT in stable and progressive myopes
AL contributed to 15% of the variance in IT, while SER accounted for 27%		
Wolsley et al. [160]	14 emmetropes (± 0.50 D), 14 mild myopes (− 0.75 to − 2.75 D), 14 moderate myopes (− 3.00 to − 5.75 D), and 14 high myopes (≤ − 6.00 D)	Reduced P1 amplitude and delayed P1 IT, with increased retinal eccentricity in high myopes
Ying et al. [144]	12 pathological myopes (AL ≥ 30.00 mm) and 24 pathological myopes (AL < 30.00 mm)	Reduced P1 amplitude, which was proportional to the neural retinal thickness in all quadrants and rings with increased AL
Shamshinova et al. [111]	46 myopes with moderate-to-high congenital myopia	Reduced P1 amplitude in all rings with increased degree of myopia and AL
Kader et al. [106]	40 emmetropes (± 0.25 D), 20 mild myopes (− 0.50 to − 3.00 D), 28 moderate myopes (− 3.25 to − 6.00 D), 40 high myopes (− 6.25 to − 15.00 D), and 40 pathological myopes (− 7.00 to − 22.00 D with 7 posterior staphyloma)	Reduced P1 amplitude and delayed P1 IT with increased degree of myopia, AL, and retinal eccentricity
Azad et al. [135]	222 emmetropes (± 0.50 D)	Maximum N1, P1, N2 amplitudes, and longest P1, N2 latencies at the fovea, which progressively decreased with increased retinal eccentricity

UNCORRECTED PROOF

Journal : Medium 10633 | Article No : 9876 | Pages : 7 | MS Code : 9876 | Dispatch : 8-7-2022

Table 2 (continued)

References	Participants	Outcomes
Park et al. [141]	30 mild myopes (− 0.50 to − 2.75 D), 25 moderate myopes (− 3.00 to − 5.75 D), 17 high myopes (− 6.00 to − 9.75 D), and 18 super high myopes (− 10.0 to − 15.0 D)	Reduced N1, P1 amplitudes and delayed P1 IT with increased degree of myopia and retinal eccentricity
Koh et al. [110]	32 myopes (≤ − 6.00 D)	Reduced P1 amplitude in the outer rings (R3-R5) with increased AL
Song et al. [142]	31 emmetropes (+ 0.75 to − 0.50 D; AL: 22 to 24 mm), 26 low-to-moderate myopes (− 0.50 to − 6.00 D; AL: 24 to 26 mm), 34 high myopes (− 6.00 to − 10.00 D; AL: 26 to 28 mm), 22 super high myopes (< − 10.00 D; AL: > 28 mm)	Reduced P1 amplitude, P1 amplitude density, and delayed P1 IT with increased degree of myopia, AL, and retinal eccentricity
Sachidanandam et al. [109]	100 eyes with AL ranging from 21.79 to 30.55 mm and SER ranging from + 0.50 to − 18.00 D	Reduced N1, P1 amplitudes and minimal delayed corresponding IT with increased AL
Ismael et al. [140]	20 emmetropes (± 0.50 D), 20 mild myopes (− 0.50 to − 3.00 D), 20 moderate myopes (− 3.00 to − 6.00 D), and 20 high myopes (< − 6.00 D)	Reduced P1 amplitude, delayed P1 latency in all rings as well as reduced N1, P1 amplitudes, delayed N1 latency in all quadrants with increased degree of myopia, AL, retinal eccentricity, and RNFL thinning
El-Gamal et al. [143]	30 emmetropes (± 0.25 D) and 30 high myopes (≤ − 5.00 D and AL > 26 mm)	Reduced N1, P1 amplitudes and corresponding IT at almost all rings and quadrants in high myopes, which was proportional to increased AL<br>P1 responses were more affected
Nebbioso et al. [162]	24 emmetropes, 24 high myopes (< − 8.00 D and AL > 26 mm) with MF, and 24 high myopes without MF	Reduced P1 amplitude and delayed P1 IT with increased macular thickness in high myopes with MF

AL axial length, *ERG* electroretinogram, *IT* implicit time, *mfERG* multi-focal electroretinogram, *MF* myopic foveoschisis, *OPs'* oscillatory potentials, *R1–R5* ring 1 to ring 5 from center to the periphery, RNFL retinal nerve fiber layer, *SER* spherical equivalent refractive error

UNCORRECTED PROOF

Table 3 Summary of studies using global-flash mfERG (gmfERG) in myopia

References	Participants	Outcomes
Chen et al. [173]	10 emmetropes (± 0.75 D) and 14 myopes (< − 0.75 D)	Increased DC, IC amplitudes in the paracentral retina (R2 to R3) with increased degree of myopia
Ho et al. [175]	54 myopes (SER: 0.00 to − 8.13 D)	Reduced paracentral DC amplitude for the 29% and 49% contrasts in myopes<br>Reduced paracentral and peripheral IC amplitudes at all contrasts measured and for the 49% contrast, respectively, in myopes<br>SER contributed to about 14% and 16% of the variance in DC and IC amplitude, respectively
Ho et al. [177]	22 myopic children (mean age: 11 ± 1 years)	Delayed DC (R3) and IC (R2 to R5) IT at 49% contrast in children with myopia progression<br>Delayed IC IT (R1) at 96% contrast in children with myopia progression
Ho et al. [176]	26 myopic children (9–13 years) with varying degrees of myopia	Reduced central DC, IC amplitudes, and paracentral IT at 49% contrast in children with myopia progression
Ho et al. [174]	52 children (9–14 years) and 19 young adults (21–28 years) with SER ranging from 0.00 to − 5.50 D	Reduced central DC amplitude at 96% contrast in myopic children<br>Reduced paracentral IC amplitude at 49% contrast in myopic adults
Chin et al. [179]	23 emmetropes to low myopes (+ 1.00 to − 3.25 D)	Reduced DC amplitude at a low SF, which increased with increasing SF, and decreased with increasing eccentricity<br>Increased IC amplitude at all SF, which decreased with increasing eccentricity
Li et al. [178]	56 emmetropic children (± 0.50 D)	Reduced central IC amplitudes at 49% contrast with the myopic changes in SER and AL after 1 year

AL axial length, *DC* direct component, *ERG* electroretinogram, *gmfERG* global-flash multi-focal electroretinogram, *IC* induced component, *IT* implicit time, *R1–R5* ring 1 to ring 5 from center to periphery, *SER* spherical equivalent refractive error, *SF* spatial frequency

Tables 1–3: Some details of the stimuli and participants are omitted from tables. Tables 1S, 2S and 3S in the electronic supplement include the omitted information to allow clearer comparisons between studies.

Publisher's Note Springer Nature remains neutral with regard to jurisdictional claims in published maps and institutional affiliations.

UNCORRECTED PROOF

Journal : **Medium 10633**	Article No : **9876**	Pages : **7**	MS Code : **9876**	Dispatch : **8-7-2022**
